# Mechanisms of Fetal Programming in Hypertension

**DOI:** 10.1155/2012/584831

**Published:** 2012-01-27

**Authors:** John Edward Jones, Julie A. Jurgens, Sarah A. Evans, Riley C. Ennis, Van Anthony M. Villar, Pedro A. Jose

**Affiliations:** Center for Molecular Physiology Research, Children's Research Institute, Children's National Medical Center, Washington, DC 20010, USA

## Abstract

Events that occur in the early fetal environment have been linked to long-term health and lifespan consequences in the adult. Intrauterine growth restriction (IUGR), which may occur as a result of nutrient insufficiency, exposure to hormones, or disruptions in placental structure or function, may induce the fetus to alter its developmental program in order to adapt to the new conditions. IUGR may result in a decrease in the expression of genes that are responsible for nephrogenesis as nutrients are rerouted to the development of more essential organs. Fetal survival under these conditions often results in low birth weight and a deficit in nephron endowment, which are associated with hypertension in adults. Interestingly, male IUGR offspring appear to be more severely affected than females, suggesting that sex hormones may be involved. The processes of fetal programming of hypertension are complex, and we are only beginning to understand the underlying mechanisms.

## 1. Introduction

Hypertension is a powerful independent risk factor for the development of well-known cardiovascular and cerebrovascular morbidities that include myocardial infarction, stroke, atherosclerosis, and death. It is a prevalent disorder that affects close to a quarter of the adult population worldwide [1]. Although often considered as a late-onset chronic disorder, hypertension is now recognized to afflict the young population, from neonates to adolescents. While the diagnosis of hypertension in adults is based on empirically determined cut-off values for both systolic and diastolic blood pressure (BP), the diagnosis of pediatric hypertension is not as straightforward since it is based on normative data [2]. Hypertension in the young is defined as the average systolic or diastolic BP that is ≥95th percentile according to age, sex, and height on three or more occasions [2]. There are no target BP values that indicate high blood pressure in children, since BP changes with age; neonates have low BP that gradually increases with age [3]. The prevalence of pediatric hypertension is estimated at 1-2% [4] and seems to have steadily increased during the past decade, which correlates with the increase in the prevalence of obesity [5]. 

A striking concept on the etiology of hypertension that has emerged during the last few decades is what is known as fetal imprinting, or developmental programming. This concept refers to the observation that adverse environmental insults early in life, particularly during critical periods of development *in utero* and the early postnatal period, can result in silent yet long-term morphological and physiological alterations that eventually translate into disease in adulthood [6]. The correlation between adverse intrauterine conditions and subsequent cardiovascular disorders was first proposed by Barker who reported a link between the mortality rate of coronary artery disease and birth weight [7, 8]. Barker hypothesized that adaptive responses to an environmental insult during early life may persist and become harmful during adulthood when the insult is no longer present. A compelling body of work from various groups around the world has corroborated these findings and has expanded the concept to include other conditions such as type 2 diabetes, obesity, and chronic kidney disease [6]. 

The biological and molecular mechanisms involved in fetal imprinting are multiple, though not completely understood, and include low birth weight (“small baby syndrome”), glucocorticoid excess, uteroplacental deficiency, sexual dimorphism, and epigenetics.

## 2. Low Birth Weight and Hypertension

Low birth weight (LBW), defined as birth weight of <2500 g by the World Health Organization, may arise from intrauterine growth restriction (IUGR, or birth weight that is <10th percentile for gestational age) or prematurity [6], with the former exerting a stronger predisposition for disease to develop and manifest later in life [9]. Several animal models have demonstrated the inverse relationship between LBW and hypertension [10, 11], which was attributable to a deficit in nephron number. Moreover, humans with LBW have significantly higher BPs, even after correction for various modifiers such as sex, cigarette smoking, weight, and use of oral contraceptives [6]. 

Two distinct mechanisms have been proposed to explain why LBW antedates the development of adult hypertension. Numerous studies indicate that birth weight is a strong predictor of nephron number and mean glomerular volume in several racial groups [12, 13]. In humans, nephrons are formed between 28–34 weeks of gestation, after which the individual has achieved a finite nephron number endowment for life (an average of 750,000 per kidney), that is, no additional nephrons are formed thereafter [14, 15]. Postnatally, an increase in tubular length and glomerular size, which varies inversely with nephron number, is achieved [16]. IUGR curtails the formation of adequate nephron number in the growing fetus, which compromises the available filtration surface area and leads to hypertension in adults [17]. Nephron-deficient rats have been shown to exhibit sodium-dependent hypertension and albuminuria [18], while postmortem examination of kidneys from hypertensive Caucasians reveals significantly fewer glomeruli per kidney and greater glomerular volume [19]. Infants who suffered from IUGR have 30–35% reduced nephron number endowment [20]. Endothelial dysfunction in individuals with LBW is another mechanism that may explain the development of hypertension later in life [21]. The perturbation of endothelial function may be due to impaired angiogenesis during fetal development or an apparent reduction in the production or function of nitric oxide [21, 22]. 

Other factors such as increases in inflammatory cytokines, the expression of metabolic genes, and changes in blood vessel formation—all of which can contribute to high blood pressure in adults—have been detected in fetuses with IUGR [23–26]. Additionally, the expression and activity of ion transporters such as Na^+^ *∕*K^+^-ATPase [27] and sodium-hydrogen exchanger 3 [28] are increased with IUGR, which may affect how electrolyte uptake or excretion is regulated in adulthood.

## 3. Uteroplacental Insufficiency and Hypertension

In 3–10% of pregnancies in the Western world, the flow of blood from mother to fetus is inadequate, resulting in utero-placental insufficiency (UPI) [29]. UPI subjects the fetus to stressors which are not normally encountered during development, such as hypoxia, altered hormone concentrations, and decreased nutrition. These unfavorable environmental factors result in the development of conditions such as IUGR [30], which typically begins midway through pregnancy and continues through the end of gestation [31]. In the presence of UPI, the fetus must deviate from its normal developmental trajectory during this critical period of growth in order to ensure the formation of vital organs and survival in adverse conditions. Though these changes may be beneficial during early life, they often lead to deleterious conditions such as hypertension in adulthood [32–34]. 

The trafficking of nutrients, hormones, and other factors essential for the development of the kidneys and vasculature is disrupted in IUGR placentas and thus may play a role in the development of hypertension. Studies have shown that downregulation of amino acid transporters [35–40], lowered fetomaternal blood flow [35, 41], and structural abnormalities are present in IUGR placentas [42–44]. Since the placenta is the medium through which the fetus receives all factors necessary for growth, disruptions in placental structure or function may alter the development of fetal vasculature, organs, or signaling pathways in ways that predispose the fetus to later development of hypertension.

As previously mentioned, the process of nephrogenesis in humans is completed by weeks 34–36 of gestation [14, 15]. IUGR that occurs prior to the generation of the full nephron complement may result in reduced renal mass [45–47], decreased nephron and glomerular endowment, and increased protein excretion [46, 48–50]. 

IUGR may result in a decrease in the expression of genes involved in nephrogenesis [51, 52], as well as a rerouting of nutrients from the kidney to more essential organs such as the brain, adrenal glands, and heart in order to enable survival of the fetus [53, 54]. While such changes allow for fetal survival during early development, they may increase the risk of susceptibility to renal injury or malfunction during adulthood [55–57] as a result of diminished nephron number and renal size [17, 58–60]. 

Studies of IUGR in animal models have demonstrated how highly sensitive the developing kidney is to perturbations of the intrauterine environment, especially during the early stages of nephrogenesis. Maternal dietary manipulation is commonly employed to induce IUGR during fetal development [46, 47] which results in reduced nephrogenesis, suppression of the renin-angiotensin system (RAS), and hypertension in the adult animal. Significantly, the timing of the induction of fetal IUGR is directly related to the outcome in the adult. The greatest impact on adult blood pressure is observed when IUGR coincides with the period of nephrogenesis. This indicates the importance of kidney morphology in the programming of blood pressure in the adult [49].

## 4. Sexual Dimorphism and Hypertension

Sex hormones may have a regulatory effect on the development of hypertension in adult IUGR offspring. While both male and female IUGR offspring are hypertensive early in life, only males remain hypertensive into adulthood. Many studies have shown that estrogen may play a protective role against the development of hypertension in female IUGR offspring [61]. This is supported by the fact that postmenopausal women are at an increased risk for hypertension. In animal models, ovariectomized female IUGR offspring become hypertensive like their male counterparts, whereas non-IUGR control females do not become hypertensive [61]. Estradiol (E2) replacement lowered blood pressure in both IUGR and control rats. It was also found that E2 levels at 16 weeks of age were not significantly different between IUGR and control offspring [61]. This seems to suggest that the protective role of estrogen does not play a direct role in regulating blood pressure, but may do so by regulating another mechanism such as the RAS. 

Testosterone seems to play a role in the stimulation of the RAS as well, which may explain the increased blood pressure in male IUGR offspring [61]. Testosterone levels were significantly higher in intact male IUGR offspring when compared to control offspring [62]. This may inappropriately stimulate the RAS and increase blood pressure [63]. Hypertension was abolished in male IUGR offspring that underwent castration, and treatment with testosterone raised their blood pressure back to hypertensive levels [62, 63]. Castration of the male control rats did not have a significant effect on blood pressure [62, 63]. The ACE inhibitor Enalapril abolished hypertension in both male and female IUGR offspring, indicating that the RAS is involved in the regulation of hypertension. Taken together, it seems that testosterone acts as a stimulant of the RAS, which increases blood pressure, while estrogen acts as an inhibitor, lowering blood pressure in females. 

Maternal diet has been shown to have a large impact on the fetal programming of hypertension, although this too has a clear sexual dimorphism. Males appear to be much more sensitive to insult *in utero* than their female counterparts [64]. Studies show that in cases of moderate fetal insult due to a protein-restricted or global-restricted diet, male IUGR offspring developed and remained hypertensive while their female counterparts seemed to be protected [16, 64]. Female IUGR offspring only responded with an increase in blood pressure to severe insult during development [64]. There could be two reasons to explain this dichotomy. One may be the influence of the sex hormones testosterone and estrogen on the RAS. Increased testosterone levels in male IUGR offspring may explain their increased sensitivity to hypertension, while the effect of estrogen explains the protected status of female offspring. Another explanation may be “catch-up growth” of IUGR offspring. While both males and females exhibit low birth weight as a result of reduced uterine perfusion, male IUGR offspring undergo catch-up growth [16, 64, 65]. This causes male IUGR offspring to have an above-average body mass as adults, which may be a factor in the development of hypertension [64].

## 5. Glucocorticoids and Hypertension

Glucocorticoids are potent regulators of fetal growth and have been associated with long-term effects on the development and morphogenesis of the growing fetus. Corticosteroid therapy for neonates at risk of preterm delivery and respiratory distress syndrome (RDS) has been the standard of treatment since the landmark paper by Liggins and Howie in 1972 [66]. Some pathophysiological effects of glucocorticoid excess are hypertension, impaired sugar metabolism, and abnormalities in neuroendocrine responses [67–69]. 

There are two ways by which an elevation of glucocorticoids may occur* in utero*. First, suboptimal placental or maternal nutrient supply results in increased glucocorticoid levels, which restrict fetal growth and program permanent changes in the cardiovascular, endocrine, and metabolic systems [70]. Second, exposure to exogenous glucocorticoids may occur, such as in neonates treated with glucocorticoids for respiratory-related disorders. Antenatal administration of exogenous or increased endogenous glucocorticoids results in LBW [71] and alters the maturation of a variety of organs. 

Glucocorticoids are lipophilic molecules that cross the placenta. Fetal glucocorticoid levels are much lower than maternal levels. In order to maintain this difference, the placenta is saturated with the enzyme 11-*β*-hydroxysteroid dehydrogenase type 2 (11-*β*-HSD2), which rapidly inactivates glucocorticoids to their inert 11-keto forms (cortisone, 11-dehydrocorticosterone) [72]. This process reduces fetal exposure to maternally active glucocorticoids. If the fetus is genetically deficient in the 11-*β*-HSD2, it may exhibit LBW and metabolic disorders. For example, heterozygous individuals carrying the deleterious 11-*β*-HSD2 gene allele had extremely low birth weights with respect to siblings who were homozygous for the wild-type gene and born with normal weights [69]. Studies in rats show that a decrease in 11-*β*-HSD2 activity results in LBW and hypertension in the adult animal [73]. 

The exact mechanism of action for glucocorticoids and the pathogenesis of hypertension have been characterized in several physiological systems. Increased production of reactive oxygen species, the activation of the RAS, and impairment of nephron development are all associated with increased sodium retention in neonates exposed to glucocorticoids [68, 74]. Research has found a critical period in early fetal development during which glucocorticoid treatment will lead to hypertension; soon after this time period, most of the organs will have developed, and thus glucocorticoid exposure will not have as profound an effect on their morphogenesis. In one study, offspring of pregnant ewes exposed to glucocorticoids developed impaired renal function and glomerular filtration rate. In two-month-old female offspring of these glucocorticoid-treated ewes, the *α*-, *β*-, *γ*-subunits of Na^+^/K^+^-ATPase were upregulated in the kidney, indicating a potential mechanism of action for development of hypertension [75]. 

Genetic predisposition can also play a role in the alteration of the glucocorticoid receptor function. The 23 K variant of the R23K SNP of the glucocorticoid receptor has been shown to protect against neonatal development of insulin resistance and growth failure. This has been shown by a study that evaluated the relative sensitivity of an individual to cortisol in a cohort of 249 19-year-old subjects who were born at less than 32 weeks gestational age. The investigators determined that genomic polymorphisms may play a role in the sensitivity of neonates to glucocorticoid exposure and may have permanent effects as a result of fetal programming [76]. Mutations in the 11-*β*-HSD2 have been linked with very low birth weights in patients with apparent mineralocorticoid excess, leading to juvenile hypertension [74].

## 6. Fetal Programming and Epigenetics

Epigenetic modification of DNA by methylation occurs primarily at CpG dinucleotides and serves as a mechanism of gene silencing. Studies using rodent models of maternal protein restriction during gestation have shown that reduced methylation of genes occurs in the offspring [77, 78]. Using a similar system, Bogdarina et al. [79] demonstrated decreased methylation at the proximal promoter region of the angiotensin receptor, type b gene (*Agtr1b*), which correlated with increased expression of the receptor in the adrenal gland of the offspring. *Agtr1b*, however, is not found in humans. Nevertheless, these results suggest a link between IUGR and epigenetic modification of genes related to the regulation of blood pressure [78]. More recently, Bogdarina et al. [80] found that treating pregnant rats with metyrapone, an 11 *β*-hydroxylase inhibitor, during the first two weeks of pregnancy normalized the methylation of the promoter region of the *Agtr1b*, reduced the expression of the Agtr1b receptor, and prevented the development of hypertension in the offspring. While few of these types of studies have been conducted, the results are exciting and further investigation is certainly warranted.

## 7. Conclusion

Twenty years ago, Hales and Barker proposed that events that occur in the early fetal environment may be linked to long-term health and lifespan consequences in the adult [81]. Numerous subsequent studies have largely supported their hypothesis and serve to illustrate the overarching complexity of the maternal-fetal-environmental interaction. While this review was necessarily limited in scope to only the effect of IUGR on hypertension, the reader should be made aware that this is but one component of a constellation of metabolic disorders that may arise as a result of a severe perturbation of the fetal environment.

The focus of most of the studies reviewed has been the impact of IUGR on the adult animal. We would suggest that additional studies be devoted to elucidating the mechanisms employed by the fetus in order to rapidly adapt to its altered environment. It is by better understanding these mechanisms that we may be able to develop interventional strategies that may prevent the onset of disease in the adult.

## References

[1] Staessen JA, Kuznetsova T, Stolarz K (2003). Hypertension prevalence and stroke mortality across populations. *Journal of the American Medical Association*.

[2] Falkner B, Daniels SR, Flynn JT (2004). The fourth report on the diagnosis, evaluation, and treatment of high blood pressure in children and adolescents. *Pediatrics*.

[3] Guignard JP (1986). Hypertension in the neonate. *Clinical and Experimental Hypertension*.

[4] Jones JE, Jose PA (2005). Hypertension in young children and neonates. *Current Hypertension Reports*.

[5] Din-Dzietham R, Liu Y, Bielo MV, Shamsa F (2007). High blood pressure trends in children and adolescents in national surveys, 1963 to 2002. *Circulation*.

[6] Zandi-Nejad K, Luyckx VA, Brenner BM (2006). Adult hypertension and kidney disease: the role of fetal programming. *Hypertension*.

[7] Barker DJP (1998). *in utero* programming of chronic disease. *Clinical Science*.

[8] Barker DJP (1990). The fetal and infant origins of adult disease. *British Medical Journal*.

[9] Yiu V, Buka S, Zurakowski D, McCormick M, Brenner B, Jabs K (1999). Relationship between birthweight and blood pressure in childhood. *American Journal of Kidney Diseases*.

[10] Manning J, Vehaskari VM (2001). Low birth weight-associated adult hypertension in the rat. *Pediatric Nephrology*.

[11] Poladia DP, Kish K, Kutay B, Bauer J, Baum M, Bates CM (2006). Link between reduced nephron number and hypertension: studies in a mutant mouse model. *Pediatric Research*.

[12] Mañalich R, Reyes L, Herrera M, Melendi C, Fundora I (2000). Relationship between weight at birth and the number and size of renal glomeruli in humans: a histomorphometric study. *Kidney International*.

[13] Hughson M, Farris AB, Douglas-Denton R, Hoy WE, Bertram JF (2003). Glomerular number and size in autopsy kidneys: the relationship to birth weight. *Kidney International*.

[14] Nigam SK, Aperia AC, Brenner BM, Brenner BM (1996). Development and maturation of the kidney. *Brenner and Rector’s The Kidney*.

[15] Puddu M, Fanos V, Podda F, Zaffanello M (2009). The kidney from prenatal to adult life: perinatal programming and reduction of number of nephrons during development. *American Journal of Nephrology*.

[16] Simeoni U, Ligi I, Buffat C, Boubred F (2011). Adverse consequences of accelerated neonatal growth: cardiovascular and renal issues. *Pediatric Nephrology*.

[17] Brenner BM, Garcia DL, Anderson S (1988). Glomeruli and blood pressure. Less of one, more the other?. *American Journal of Hypertension*.

[18] Sanders MW, Fazzi GE, Janssen GMJ, Blanco CE, De Mey JGR (2005). High sodium intake increases blood pressure and alters renal function in intrauterine growth-retarded rats. *Hypertension*.

[19] Keller G, Zimmer G, Mall G, Ritz E, Amann K (2003). Nephron number in patients with primary hypertension. *New England Journal of Medicine*.

[20] Hinchliffe SA, Lynch MRJ, Sargent PH, Howard CV, Van Velzen D (1992). The effect of intrauterine growth retardation on the development of renal nephrons. *British Journal of Obstetrics and Gynaecology*.

[21] Goodfellow J, Bellamy MF, Gorman ST (1998). Endothelial function is impaired in fit young adults of low birth weight. *Cardiovascular Research*.

[22] Leeson CPM, Kattenhorn M, Morley R, Lucas A, Deanfield JE (2001). Impact of low birth weight and cardiovascular risk factors on endothelial function in early adult life. *Circulation*.

[23] Street ME, Seghini P, Feini S (2006). Changes in interleukin-6 and IGF system and their relationships in placenta and cord blood in newborns with fetal growth restriction compared with controls. *European Journal of Endocrinology*.

[24] Gauster M, Hiden U, Blaschitz A (2007). Dysregulation of placental endothelial lipase and lipoprotein lipase in intrauterine growth-restricted pregnancies. *Journal of Clinical Endocrinology and Metabolism*.

[25] Jarvenpaa J, Vuoristo JT, Savolainen ER, Ukkola O, Vaskivuo T, Ryynanen M (2007). Altered expression of angiogenesis-related placental genes in pre-eclampsia associated with intrauterine growth restriction. *Gynecological Endocrinology*.

[26] Thornburg KL, O’Tierney PF, Louey S (2010). Review: the placenta is a programming agent for cardiovascular disease. *Placenta*.

[27] Bertram C, Trowern AR, Copin N, Jackson AA, Whorwood CB (2001). The maternal diet during pregnancy programs altered expression of the glucocorticoid receptor and type 2 11*β*-hydroxysteroid dehydrogenase: potential molecular mechanisms underlying the programming of hypertension *in utero*. *Endocrinology*.

[28] Dagan A, Gattineni J, Cook V, Baum M (2007). Prenatal programming of rat proximal tubule Na^+^/H^+^ exchanger by dexamethasone. *American Journal of Physiology*.

[29] Witlin AG, Sibai BM (1997). Hypertension in pregnancy: current concepts of preeclampsia. *Annual Review of Medicine*.

[30] Economides DL, Nicolaides KH (1989). Blood glucose and oxygen tension levels in small-for-gestational-age fetuses. *American Journal of Obstetrics and Gynecology*.

[31] Smith GCS (2004). First trimester origins of fetal growth impairment. *Seminars in Perinatology*.

[32] Barker DJP, Osmond C, Golding J, Kuh D, Wadsworth MEJ (1989). Growth *in utero*, blood pressure in childhood and adult life, and mortality from cardiovascular disease. *British Medical Journal*.

[33] Olofsson P, Laurini RN, Marsal K (1993). A high uterine artery pulsatility index reflects a defective development of placental bed spiral arteries in pregnancies complicated by hypertension and fetal growth retardation. *European Journal of Obstetrics Gynecology and Reproductive Biology*.

[34] Gluckman PD, Hanson MA, Cooper C, Thornburg KL (2008). Effect of *in utero* and early-life conditions on adult health and disease. *New England Journal of Medicine*.

[35] Laurin J, Lingman G, Marsal K, Persson PH (1987). Fetal blood flow in pregnancies complicated by intrauterine growth retardation. *Obstetrics and Gynecology*.

[36] Dicke JM, Henderson GI (1988). Placental amino acid uptake in normal and complicated pregnancies. *American Journal of the Medical Sciences*.

[37] Mahendran D, Donnai P, Glazier JD, D’Souza SW, Boyd RDH, Sibley CP (1993). Amino acid (system A) transporter activity in microvillous membrane vesicles from the placentas of appropriate and small for gestational age babies. *Pediatric Research*.

[38] Glazier JD, Cetin I, Perugino G (1997). Association between the activity of the system A amino acid transporter in the microvillous plasma membrane of the human placenta and severity of fetal compromise in intrauterine growth restriction. *Pediatric Research*.

[39] Norberg S, Powell TL, Jansson T (1998). Intrauterine growth restriction is associated with a reduced activity of placental taurine transporters. *Pediatric Research*.

[40] Jansson T, Scholtbach V, Powell TL (1998). Placental transport of leucine and lysine is reduced in intrauterine growth restriction. *Pediatric Research*.

[41] Nylund L, Lunell NO, Lewander R, Sarby B (1983). Uteroplacental blood flow index in intrauterine growth retardation of fetal or maternal origin. *British Journal of Obstetrics and Gynaecology*.

[42] Müntefering H, Wysocki M, Rastorguev E, Gerein V (2004). Placenta in gestational hypertension. *Pathologe*.

[43] Battistelli M, Burattini S, Pomini F, Scavo M, Caruso A, Falcieri E (2004). Ultrastructural study on human placenta from intrauterine growth retardation cases. *Microscopy Research and Technique*.

[44] Sahin Z, Acar N, Ozbey O, Ustunel I, Demir R (2011). Distribution of Notch family proteins in intrauterine growth restriction and hypertension complicated human term placentas. *Acta Histochemica*.

[45] Merlet-Benichou C, Gilbert T, Muffat-Joly M, Lelievre-Pegorier M, Leroy B (1994). Intrauterine growth retardation leads to a permanent nephron deficit in the rat. *Pediatric Nephrology*.

[46] Langley-Evans SC, Welham SJM, Jackson AA (1999). Fetal exposure to a maternal low protein diet impairs nephrogenesis and promotes hypertension in the rat. *Life Sciences*.

[47] Vehaskari VM, Aviles DH, Manning J (2001). Prenatal programming of adult hypertension in the rat. *Kidney International*.

[48] Woods LL (2000). Fetal origins of adult hypertension: a renal mechanism?. *Current Opinion in Nephrology and Hypertension*.

[49] Woods LL, Ingelfinger JR, Nyengaard JR, Rasch R (2001). Maternal protein restriction suppresses the newborn renin-angiotensin system and programs adult hypertension in rats. *Pediatric Research*.

[50] Schreuder MF, Nyengaard JR, Fodor M, Van Wijk JAE, Delemarre-Van De Waal HA (2005). Glomerular number and function are influenced by spontaneous and induced low birth weight in rats. *Journal of the American Society of Nephrology*.

[51] Moritz KM, Wintour EM, Black MJ, Bertram JF, Caruana G (2008). Factors influencing mammalian kidney development: implications for health in adult life. *Advances in Anatomy, Embryology, and Cell Biology*.

[52] Abdel-Hakeem AK, Henry TQ, Magee TR (2008). Mechanisms of impaired nephrogenesis with fetal growth restriction: altered renal transcription and growth factor expression. *American Journal of Obstetrics and Gynecology*.

[53] Barker DJP, Hales CN, Fall CHD, Osmond C, Phipps K, Clark PMS (1993). Type 2 (non-insulin-dependent) diabetes mellitus, hypertension and hyperlipidaemia (syndrome X): relation to reduced fetal growth. *Diabetologia*.

[54] Silver LE, Decamps PJ, Korst LM, Platt LD, Castro LC (2003). Intrauterine growth restriction is accompanied by decreased renal volume in the human fetus. *American Journal of Obstetrics and Gynecology*.

[55] Schreuder MF, Van Wijk JAE, Fodor M, Delemarre-van de Waal HA (2007). Influence of intrauterine growth restriction on renal function in the adult rat. *Journal of Physiology and Biochemistry*.

[56] Woods LL (2007). Maternal nutrition and predisposition to later kidney disease. *Current Drug Targets*.

[57] Ojeda NB, Grigore D, Alexander BT (2008). Developmental programming of hypertension: insight from animal models of nutritional manipulation. *Hypertension*.

[58] Speth RC, Husain A (1988). Distribution of angiotensin-converting enzyme and angiotensin II-receptor binding sites in the rat ovary. *Biology of Reproduction*.

[59] Von Lutterotti N, Camargo MJF, Mueller FB, Timmermans PBMWM, Laragh JH (1991). Angiotensin II receptor antagonist markedly reduces mortality in salt-loaded Dahl S rats. *American Journal of Hypertension*.

[60] Krebs LT, Hanesworth JM, Sardinia MF, Speth RC, Wright JW, Harding JW (2000). A novel angiotensin analog with subnanomolar affinity for angiotensin- converting enzyme. *Journal of Pharmacology and Experimental Therapeutics*.

[61] Ojeda NB, Grigore D, Robertson EB, Alexander BT (2007). Estrogen protects against increased blood pressure in postpubertal female growth restricted offspring. *Hypertension*.

[62] Ojeda NB, Grigore D, Yanes LL (2007). Testosterone contributes to marked elevations in mean arterial pressure in adult male intrauterine growth restricted offspring. *American Journal of Physiology*.

[63] Ojeda NB, Royals TP, Black JT, Dasinger JH, Johnson JM, Alexander BT (2010). Enhanced sensitivity to acute angiotensin II is testosterone dependent in adult male growth-restricted offspring. *American Journal of Physiology*.

[64] Grigore D, Ojeda NB, Alexander BT (2008). Sex differences in the fetal programming of hypertension. *Gender Medicine*.

[65] de Gusmão Correia ML, Volpato AM, Aguila MB, Mandarim-de-Lacerda CA Developmental origins of health and disease: experimental and human evidence of fetal programming for metabolic syndrome.

[66] Liggins GC, Howie RN (1972). A controlled trial of antepartum glucocorticoid treatment for prevention of the respiratory distress syndrome in premature infants. *Pediatrics*.

[67] Villar VA, Liu T, Jose PA (2010). Recent trends in pediatric hypertension research. *Le Journal Médical Libanais*.

[68] Dagan A, Habib S, Gattineni J, Dwarakanath V, Baum M (2009). Prenatal programming of rat thick ascending limb chloride transport by low-protein diet and dexamethasone. *American Journal of Physiology*.

[69] Seckl JR, Cleasby M, Nyirenda MJ (2000). Glucocorticoids, 11*β*-hydroxysteroid dehydrogenase, and fetal programming. *Kidney International*.

[70] Edwards LJ, Coulter CL, Symonds ME, McMillen IC (2001). Prenatal undernutrition, glucocorticoids and the programming of adult hypertension. *Clinical and Experimental Pharmacology and Physiology*.

[71] French NP, Hagan R, Evans SF, Godfrey M, Newnham JP (1999). Repeated antenatal corticosteroids: size at birth and subsequent development. *American Journal of Obstetrics and Gynecology*.

[72] Brown RW, Chapman KE, Kotelevtsev Y (1996). Cloning and production of antisera to human placental 11*β*-hydroxysteroid dehydrogenase type 2. *Biochemical Journal*.

[73] Benediktsson R, Lindsay RS, Noble J, Seckl JR, Edwards CRW (1993). Glucocorticoid exposure *in utero*: new model for adult hypertension. *The Lancet*.

[74] Gwathmey TM, Shaltout HA, Rose JC, Diz DI, Chappell MC (2011). Glucocorticoid-induced fetal programming alters the functional complement of angiotensin receptor subtypes within the kidney. *Hypertension*.

[75] Moritz KM, de Matteo R, Dodic M (2011). Prenatal glucocorticoid exposure in the sheep alters renal development *in utero*: implications for adult renal function and blood pressure control. *American Journal of Physiology*.

[76] Finken MJJ, Meulenbelt I, Dekker FW (2007). The 23K variant of the R23K polymorphism in the glucocorticoid receptor gene protects against postnatal growth failure and insulin resistance after preterm birth. *Journal of Clinical Endocrinology and Metabolism*.

[77] Lillycrop KA, Phillips ES, Jackson AA, Hanson MA, Burdge GC (2005). Dietary protein restriction of pregnant rats induces and folic acid supplementation prevents epigenetic modification of hepatic gene expression in the offspring. *Journal of Nutrition*.

[78] Lillycrop KA, Slater-Jefferies JL, Hanson MA, Godfrey KM, Jackson AA, Burdge GC (2007). Induction of altered epigenetic regulation of the hepatic glucocorticoid receptor in the offspring of rats fed a protein-restricted diet during pregnancy suggests that reduced DNA methyltransferase-1 expression is involved in impaired DNA methylation and changes in histone modifications. *British Journal of Nutrition*.

[79] Bogdarina I, Welham S, King PJ, Burns SP, Clark AJL (2007). Epigenetic modification of the renin-angiotensin system in the fetal programming of hypertension. *Circulation Research*.

[80] Bogdarina I, Haase A, Langley-Evans S, Clark AJL (2010). Glucocorticoid effects on the programming of AT1b angiotensin receptor gene methylation and expression in the rat. *PLoS One*.

[81] Hales CN, Barker DJP (1992). Type 2 (non-insulin-dependent) diabetes mellitus: the thrifty phenotype hypothesis. *Diabetologia*.

